# The extent of distal intramural spread of colorectal cancer cell study of it's relationship with histological grading, stage of disease and CEA level

**DOI:** 10.1016/j.amsu.2021.102227

**Published:** 2021-03-24

**Authors:** Arif Zuhan, Ignatius Riwanto, Devia Eka Listiana, Fathul Djannah, Rohadi Muhammad Rosyidi

**Affiliations:** a Digestive Surgery Subdivision, Department of Surgery Medical Faculty of Mataram University, West Nusa Tenggara General Hospital, Mataram, Indonesia; b Department of Digestive Surgery, Faculty of Medicine, Diponegoro University/Dr.Kariadi General Hospital, Indonesia; c Department of Anatomical Pathology, Faculty of Medicine, Diponegoro University/Dr.Kariadi General Hospital, Indonesia; d Department of Anatomical Pathology, Medical Faculty of Mataram University, Mataram, Indonesia; e Department of Neurosurgery Medical Faculty of Mataram University, West Nusa Tenggara General Hospital, Mataram, Indonesia

**Keywords:** Colorectal cancer, Distal intramural spread, Histologic grading, Stage of disease, CEA

## Abstract

**Background:**

The free margin of distal resection is an attempt to prevent local recurrence of the tumor and prolong survival. The recommended length of distal resection margin are varied among the researchers. This study was done to know the correlation between extents of distal intramural spread (DIS) and histology grading, stage and CEA levels of colorectal cancer.

**Methods:**

The design of the study was a cross sectional. Sample was patients diagnosed with colon or rectal adenocarcinoma in the period of September 2017–March 2018 and underwent resection at Dr.Kariadi Hospital. Resected fresh tissue tumors were directly measured for the distal resection margin and histopathologic examination done by anatomical pathologists. This study has been approved by the ethics committee of Dr.Kariadi Hospital/Faculty of Medicine Diponegoro University. The relationship between DIS length to histology grading, tumor stage and CEA level were analyzed using Spearman's correlation test.

**Results:**

The subjects of this study were 26 patients with colorectal cancer consisted of 15 men and 11 women. The average age of the patients was 53,04 years. The locations of the tumor were 17 in the rectum and 9 in the colon. The length DIS were between 1,07 and 11,49 mm. The longer DIS were occurred when the grading histology worsens, the tumor stage increases and the higher CEA levels with correlation coefficient were r = 0,77 (p < 0,001); r = 0,66 (p < 0,001) and r = 0,44 (p = 0,024) respectively. For the rectal location, the DIS length range were 0,28–10,36 mm. The longer DIS when grading histology worsens r = 0,59 (p = 0,012) and an increased tumor stage r = 0,73 (p = 0,001). The DIS length of the rectum was not proven to correlate with elevated CEA levels r = 0,14 (p = 0,588).

**Conclusion:**

Histological grading, tumor stage and CEA levels can be predictors of distal intramural spread (DIS) colorectal cancer. The strongest correlation were between DIS and histologic grading. Thus, in mid and lower third of the rectal cancer, the histologic grade examination is strongly recommended. Based on this study, it is recommended that in rectal cancer undergoing sphincter preserving surgery distal resection sould be more than 2 cm from the tumor margin.

## Highlights

• This study was done to know the correlation between extents of distal intramural spread (DIS) and histology grading, stage and CEA levels of colorectal cancer.
• Histological grading, tumor stage and CEA levels can be predictors of distal intramural spread (DIS) colorectal cancer.
• Based on this study, it is recommended that in rectal cancer undergoing sphincter preserving surgery distal resection sould be more than 2 cm from the tumor margin.

## 1 Introduction

The main key to successful treatment of colorectal carcinoma is to diagnose of carcinoma in the early stage, so that therapy can be performed by curative surgery. Surgical treatment is most effective when done in a disease that is still localized. When metastasis occurs, the prognosis is poor and the survival rate decreases. Surgery is the first line treatment for nonmetastatic colorectal carcinoma, the ultimate goal is the limit of tumor resection without the remaining residue of cancer cells [1].

The tumor-free distal margin resection is an attempt to prevent local recurrence of tumors and prolong survival. The recommended limit of distal resection varies between one and another researchers. Krzysztof Bujko et al., 2012 from the Gastrointestinal Section of Cancer Warsaw Poland made a study which concluded that there was no significant difference between <1 cm and >1 cm resection margin groups of the tumor against local recurrence and survival rates [2]. Other researchers Williams et al., 1983; Shirozou et al., 1995; Mezhir et al., 2005; Kwok et al., 1996 reported that intramural spread to the distal of the tumor appears to be still positive within 1 cm. Based on the above finding, they recommend in cases of rectosigmoid cancer who will be performed Anterior Resection action, margin resection should be > 1 cm as an acceptable minimum threshold. Ono et al. Reported that the maximum length of distal distribution of cancer cells in colorectal was 2.4 cm, and it was concluded that the optimal distal margin clearance was 3 cm. The current general rule in Japan for colorectal cancer suggests that resection margin for tumor above the peritoneal reflection should be 3 cm, and for below peritoneal reflection requires a margin of 2 cm. Shimada et al., 2011 recommended increase of 1–2 cm beyond the recommended distal resection margin from current Japanese general rules may contribute to improve local control [3].

In Indonesia, the recommendation of distal resection limit has been revised several times, from 5 cm to 2 cm. It is based on study in 2016, showed that 81%–95% of carcinomas do not spread or intramural extensions exceed 1 cm, rectum cancer extension of more than 1 cm is always at an advanced stage (poor differentiation) or there has been a distant metastasis [4].

Based on various variations of long resection margins from several studies in other countries, and there is no research on this topic in Indonesia, we did a study regarding correlation of between distal intramural spread in one hand and histological grading, stage of tumour and CEA level on other hand. The result of this study is expected to give scientific recommendation regarding the save distal resection margin.

## 2 Methods

The design of this study was cross sectional. In this study, samples were patients who were diagnosed with colon and rectal adenocarcinoma in September 2017–March 2018 and performed tumor resection at Dr. Kariadi Hospital, which is supported by examination and histopathology data, according to The STROCSS 2019 Guideline [5]. The data were collected from resected tumor and then measured directly to the distal resection limit on the fresh tissue and examined histopathologically in collaboration with the Anatomy Pathology department. This research has received approval from the Ethics Committee of the Faculty of Medicine Diponegoro University/Dr.Kariadi Hospital Semarang. Data collected, were analyzed to know the correlation between DIS length of cancer cells on one hand and histological grading, tumor stage and CEA levels on the other hand. Data were analyzed by Spearman correlation test.

### 2.1 Specimen treatment

**Fig. 1:**
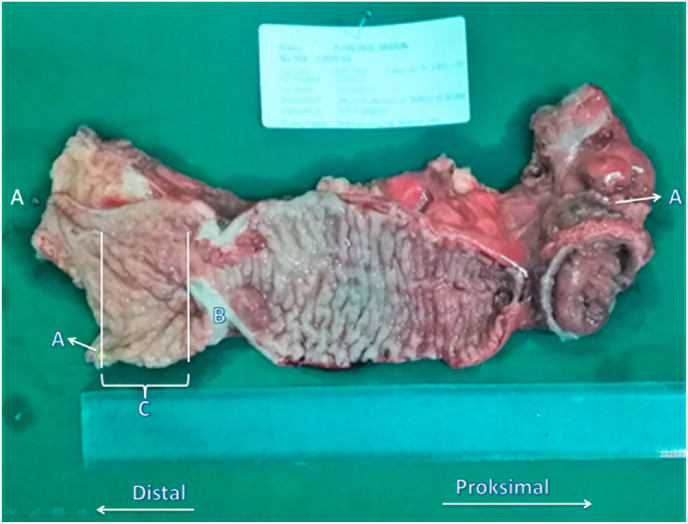
Fresh specimen middle third adeno Ca recti. A. Needle pin, B. Tumor Mass, C. Length distal resection margin.

The received specimen is not fixed first. After the tumor mass is resected, the time is recorded, then specimens are cleaned with running water from feces and blood. The specimen is placed on a cork board, opened longitudinally, straightened without stretching, then pinned with a needle to the cork board (Fig. 1).

**Fig. 2:**
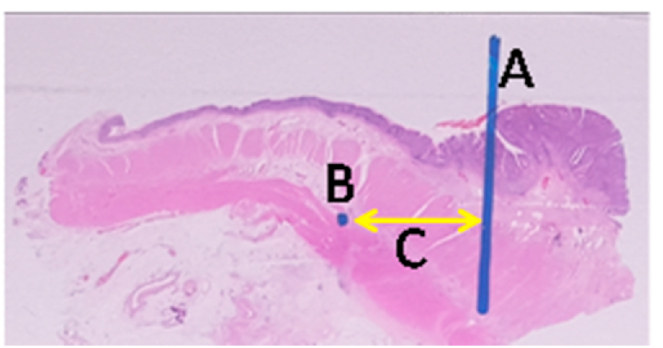
Microscopic specimen A. The distal border of the tumor is seen macroscopically (basis for measurement) B. The most distal tumor cell that seen microscopically. C. Length of Distal intramural spread.

Before it was fixed, the specimen measured the macroscopic distance of the distal resection boundary to the distal edge of the tumor, the size of the tumor and its location recorded. Specimens were fixed in 10% formalin liquids for 24–48 h.

Once fixed, the tumor distance is measured to the distal resection, cross-sectioned through the distal portion of the tumor, then made several longitudinal pieces to the limit of the distal resection. (taken maximum 5 cm from distal tumor). From the cross section, each piece is made 0.5 cm wide to distal resection. Specimens are made in paraffin blocks and HE staining is performed Specimens were assessed for type and grading histology. The distal intramural infiltration length of cancer cells is measured by a micrometer from the distal border of the tumor macroscopically [[6], [7], [8], [9]].

## 3 Result

The total subjects of this study were 26 patients with colorectal cancer consisted of 15 men and 11 women. The average age of the patients was 53.04 years. The location of the tumor consists of 17 in the rectum and 9 in the colon. Characteristics of the total sample can be seen in Table 1.

**Table 1:** Characteristics of colorectal samples.

Characteristics	n	%	Range
Sex			
Man	15	57,7	
Woman	11	42,3	
Age (years)			43,51–62,57
Treatment			
LAR	14	53,8	
Non LAR	12	46,2	
Grading			
Well	11	42,3	
Moderat	12	46,2	
Poor	3	11,5	
Tumor location			
Rectum	17	65,4	
Colon	9	34,6	
Tumor size (mm)			69,7–471,1
Range of distal resection (mm)			15,1–59,44
Distal intramural spread (mm)			1,07–11,49
CEA			2,74–12,52
Stadium			
I	2	7,7	
II A	3	11,5	
II B	4	15,4	
III B	7	26,9	
III C	5	19,2	
IV A	5	19,2	

The range length of distal intramural spread were between 1,07 and 11,49 mm. The range of distal resection margin were between 15,1 and 59,44 mm with pathologic examination result all free of tumor. The highest grading histology samples were moderate differentiated 12 cases (46.2%), well differentiated 11 cases (42.3%) and poor differentiated 3 cases (11.5%). The largest distribution of stage stages was stage III 12 cases (46,2%). Stage II 7 cases, stage IV 5 cases and stage I had 2 cases.

**Fig. 3:**
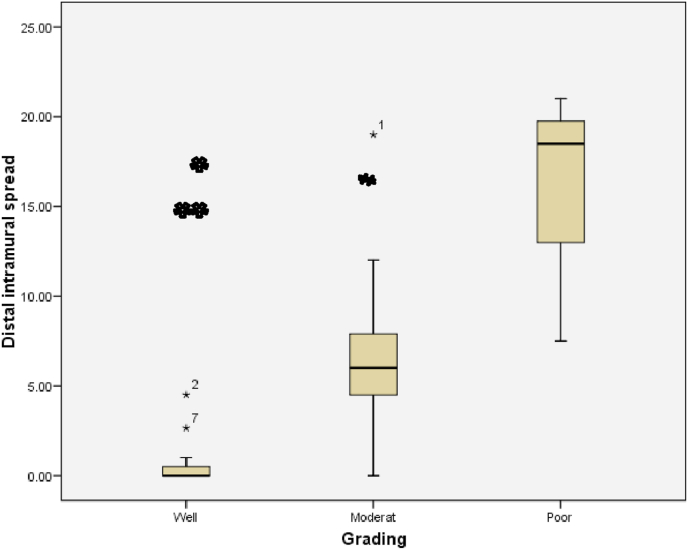
Box plot of relationship of histological grading with DIS. There were statistically significant (Kruskall Wallis p < 0,001). The different between histological grading and DIS for *Well versus moderate and **well versus poor were significantly different with p = 0,001 and p = 0,004 respectivelly, #while between moderate and poor there was not statistically different (p = 0,07).

**Fig. 4:**
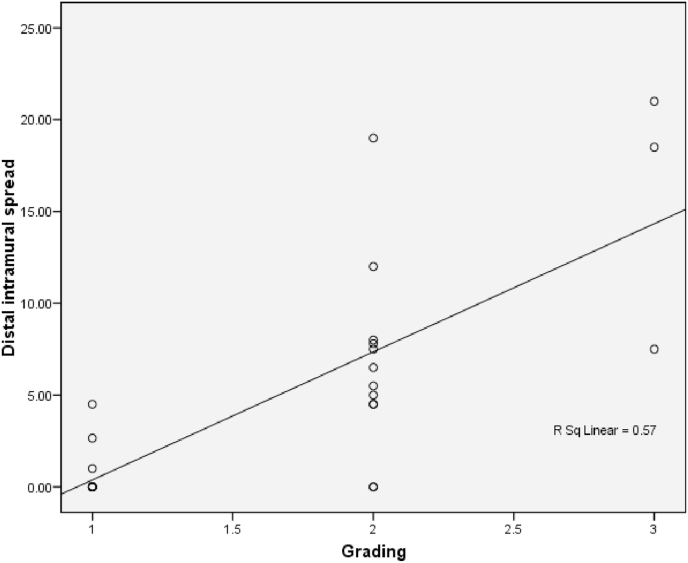
Correlation between DIS and histological grading (r = 0,771 and p < 0,001).

The relationship between histological grading and distal intramural spread can be seen from Fig. 3 and Fig. 4 (see Fig. 2). The worst histological grading the longer distal intramural spread (Fig. 3) and also there was significant strong positif correlation (Fig. 4).

**Fig. 5:**
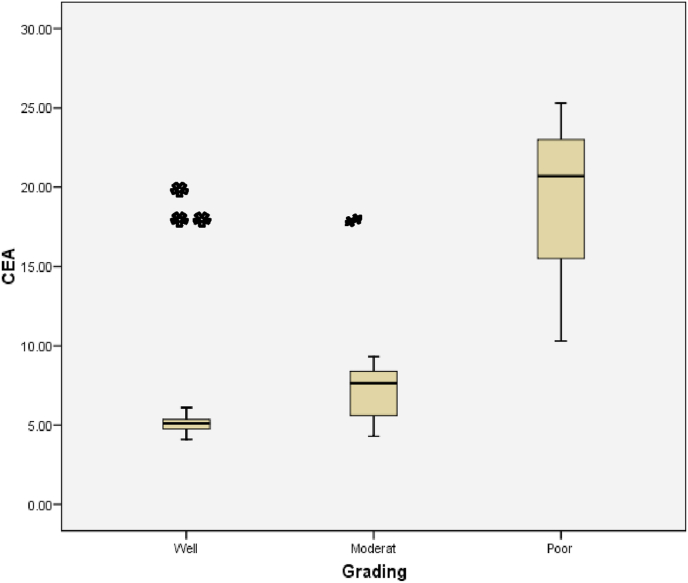
Box plot of relationship between Grade and CEA level. There were significantly differences (ANOVA p < 0,001). The difference between histological grading and CEA. *Well versus moderate was statistically different p = 0,003, while **well versus poor and #moderate versus poor were not statistically different with p = 0,161 and p = 0,209 respectivelly.

**Fig. 6:**
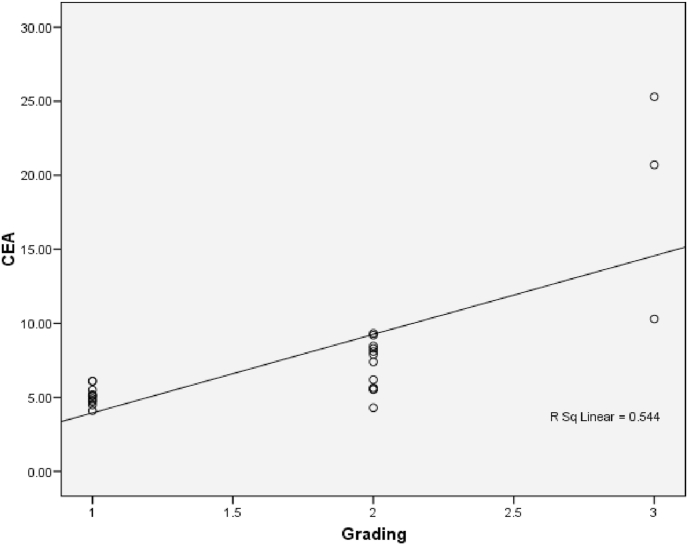
Correlation between Histological Grading and CEA (p < 0,001; r = 0,770).

The relationship between histological grading and CEA level can be seen from Figs. 3 and 4. The worst histological grading the higher CEA level (Fig. 5) and also there was significant strong positif correlation (Fig. 6).

**Fig. 7:**
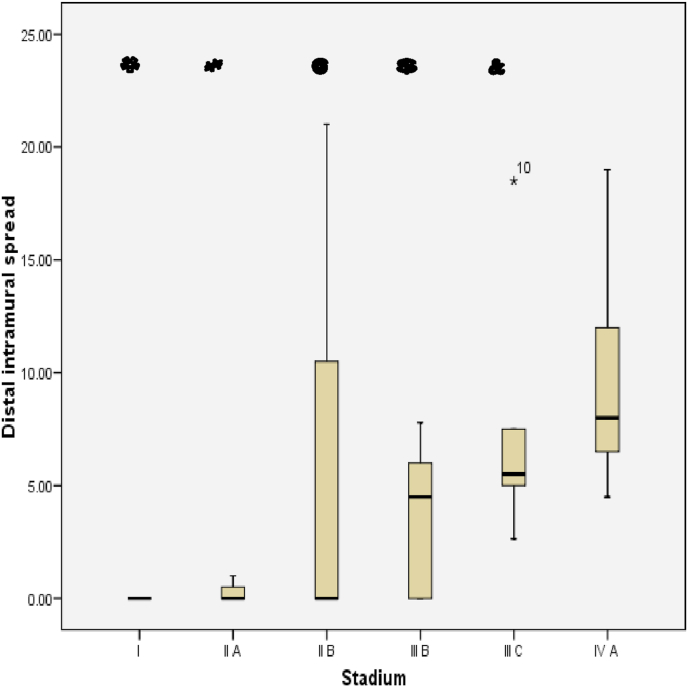
Box plot of Relationship between DIS and Stage. There were significantly differences (Kruskal Wallis, p = 0,049).

Data in colon and rectum that have been obtained, we specialize again to analyze DIS at rectal location. The following is the data characteristic of the rectum (Table 2).

**Table 2:** Characteristics of the Rectum sample.

Variable	n	%	Range
Sex			
Man	12	70,6	
Woman	5	29,4	
Age (years)			42,04–63,96
Tindakan			
LAR	14	82,4	
Non LAR	3	17,6	
Grading			
Well	5	29,4	
Moderat	11	64,7	
Poor	1	5,9	
Tumor size (mm)			18,61–313,75
Range of distal resection (mm)			16,36–30,00
Distal intramural spread (mm)			0,28–10,36
CEA			4,73–8,43
Stadium			
II A	1	5,9	
II B	1	5,9	
III B	7	41,2	
III C	4	23,5	
IV A	4	23,5	

From the rectal data obtained a sample of 17 patients with rectal cancer consisted of 12 men and 5 women. The average age of patients is 53 years. The range length of distal intramural spread were between 0,28 and 10,36 mm. The range of distal resection margin were 16,36–30,0 mm with pathologic results were all free of tumor. The highest grading histology samples were moderate differentiated 11 cases (64.7%), well differentiated 5 cases (29.4%) and poor differentiated 1 case (5.9%). The highest distribution of stage samples is stage III 11 cases (64,7%). Stage II 2 cases (11.8%), stage IV 4 cases (23.5%) and stage I did not exist.

The relationship between histological grading and distal intramural spread in rectum can be seen from Fig. 10 and Fig. 11 (see Fig. 9). The worst histological grading was not mean the longer distal intramural spread (Fig. 10) but there was significant moderate positif correlation (Fig. 11).

**Fig. 8:**
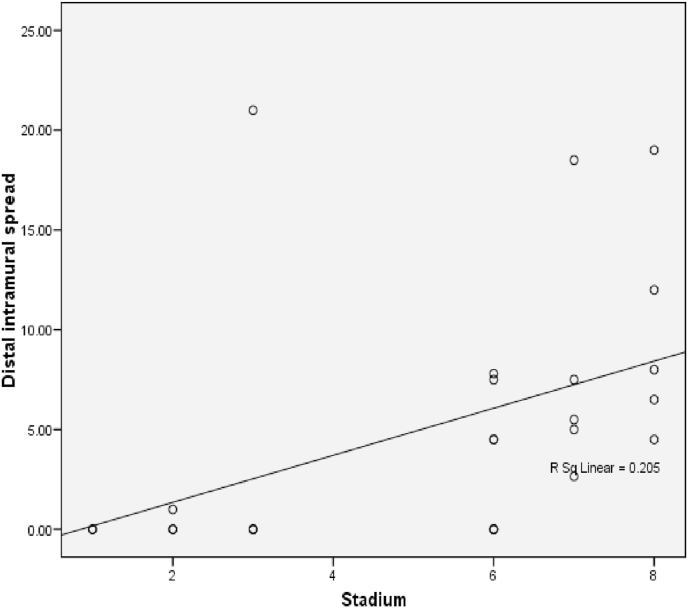
The Correlation between DIS and Stage (p < 0,001; r = 0,660). The relationship between DIS and stage can be seen from Figs. 5 and Fig. 6. The worst stage the longer DIS (Fig. 7) and also there was significant strong positif correlation (Fig. 8).

**Fig. 9:**
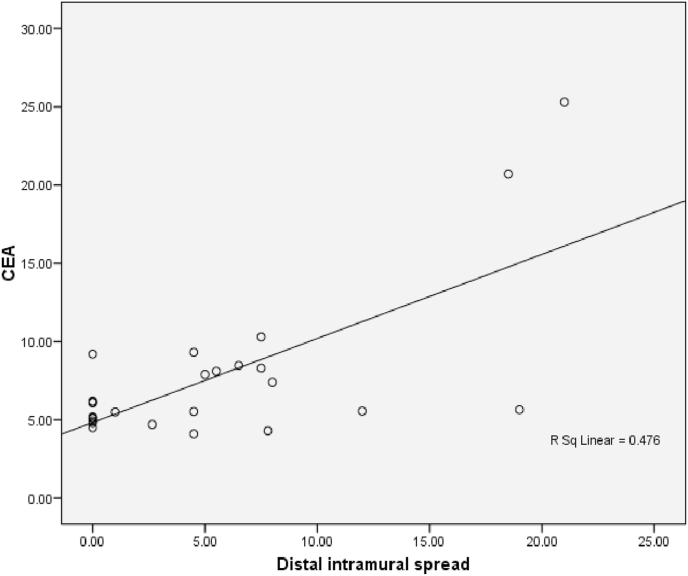
The Correlation between DIS and CEA (p = 0,024; r = 0,442). Correlation of DIS with CEA level showed a significant week positive correlation.

**Fig. 10:**
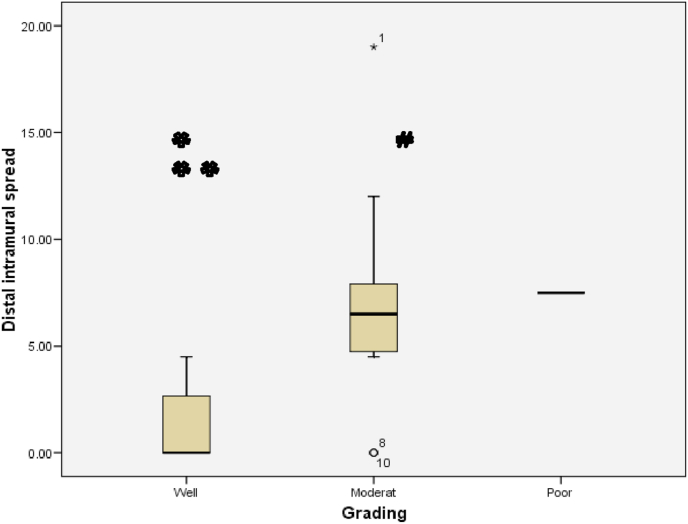
Box plot of Relationship histological grading and DIS of rectal cancer. There were statistically not significant (Kruskall Wallis p = 0,052). The different between histological grading and DIS for *well versus moderate was statistically significant (p = 0,021) while **well versus poor and # moderate versus poor were not statistically significant, p = 0,120 and p = 0,771 respectively).

**Fig. 11:**
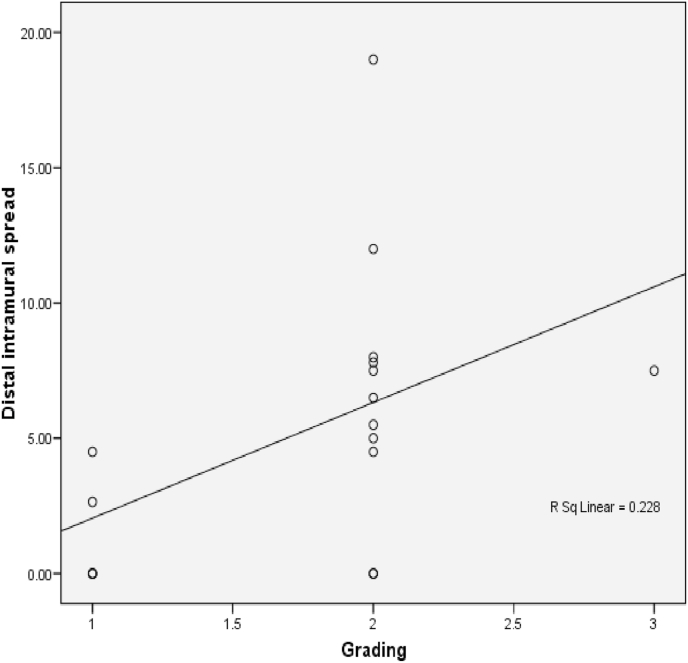
The correlation between histological grading and Distal Intramural Spread in rectal cancer (r = 0,594 and p = 0,012).

**Fig. 12:**
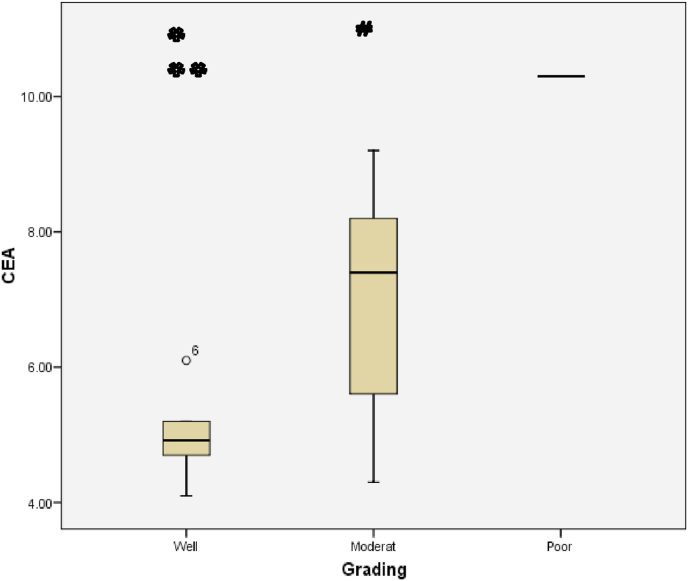
Box plot of relationship between Grade and CEA in rectal cancer. There were significantly different (Kruskal Wallis p = 0,024). The difference between histological grading and CEA,*well versus moderate were statistically significant (p = 0,020) while **well versus poor and # moderate versus poor there was not statistically significant, p = 0,143 and p = 0,111 respectively.

**Fig. 13:**
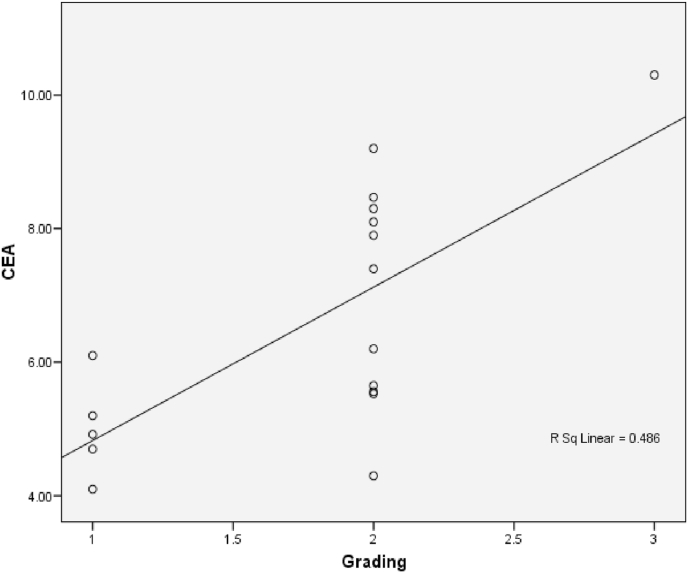
The correlation between histological grading and CEA in rectum (r = 0,677 and p = 0,003).

The relationship between histological grading and CEA level in rectum can be seen from Fig. 12 and Fig. 13. The worst histological grading the higher CEA level (Fig. 12) and also there was significant strong positif correlation (Fig. 13).

**Fig. 14:**
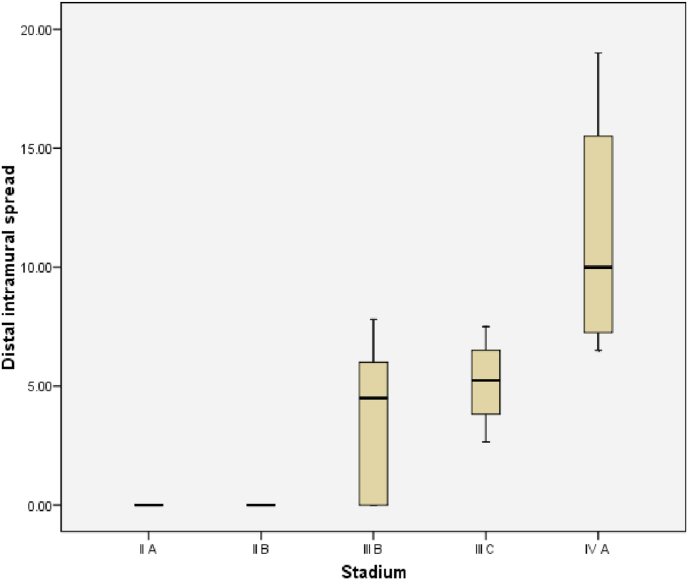
Box plot of Relationship DIS with Stage in rectum. There were not significantly different (Kruskal Wallis, p = 0,057).

**Fig. 15:**
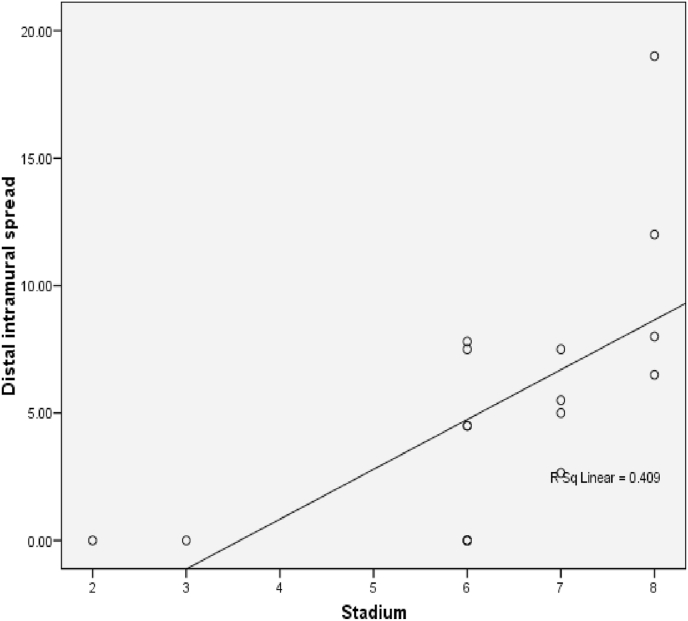
The Correlation between DIS and Stage in rectum (p = 0,001; r = 0,733).

**Fig. 16:**
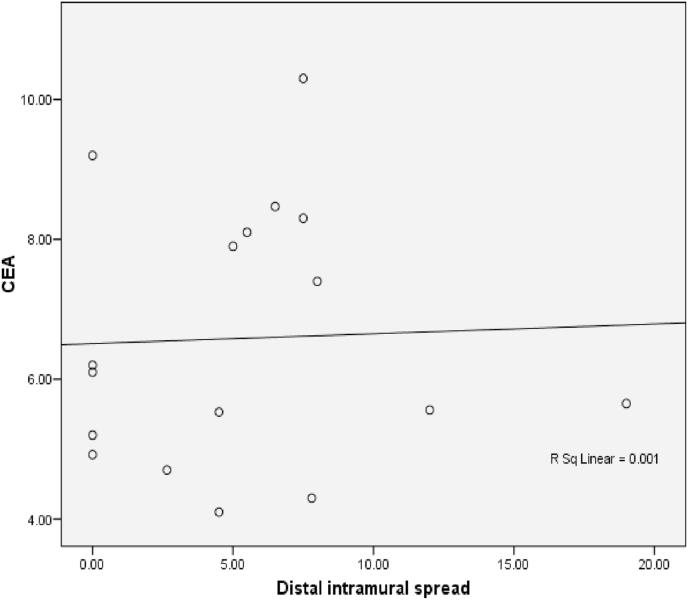
The Correlation between DIS and CEA in rectum (p = 0,588; r = 0,142).

The relationship between DIS and stage in rectum can be seen from Fig. 14 and Fig. 15. The worst stage was not mean the longer DIS (Fig. 14) but there was significant strong positif correlation (Fig. 16).

Correlation of DIS with CEA in rectum showed that there was no significant correlation.

## 4 Discussion

It was reported that histologic grade significantly affects the infiltration of cancer cells [9]. Wood et al. reported that histological gradding of the tumor was strongly associated with local invasion of tumor cells and lymph node metastasis [10]. Cancer cells corresponding to their histologic grade produced the Matrix Metalloprotein-9 (MMP-9). MMPs divide and degrade all the structural elements of the extracellular matrix (ECM) therefore plays a significant role in local infiltration associated with distal spreading (metastases). This action can affect on levels of CEA in the blood. MMP activates VEGF in angiogenesis process. In the end, the process of infiltration, metastasis and angiogenesis will establish the staging of colorectal cancer [11].

One study reported that DIS in CRC was found so vary between 3 and 58% [9]. DIS length in general was <10 mm. DIS> 10 mm is commonly present in tumors that have metastasis lymph nodes or those with advanced stages [12]. In this study, samples of cancer in the colon and rectum totally 26 patients and we found DIS in 16 samples (61.5%). Grading histology samples obtained 11 well differentiated samples (42.3%), 12 moderate samples differentiated (46.2%) and 3 poor differentiated samples (11.5%). There was a significant different between DIS with each of histological gradding in colorectal samples. The correlation of grade histology with DIS showed a significant relationship (p < 0.001) with a strong positive correlation. The longer the DIS shows the worse the grading of histology. In a well differentiated sample the range DIS length was 0–2,3 mm, in the moderate differentiated range of DIS was 1,58-11,8 mm, whereas in the poor differentiated the range of DIS was 8,49-22,8 mm. These results suggest that preoperative histology grading examination is essential to determine the action on the distal tumor resection margins. Shirouzu et al. suggests that a 10 mm distal resection margin is the appropriate clearance limit for most rectum cancers. It is mentioned that in rectum cancer extension of more than 10 mm is always at an advanced stage, differentiation is poor or there has been a distant metastasis [13].

Andreola et al. study showed that the increasing extension of DIS is associated with the type of cancer infiltrating at the tumor edge (p = 0,002) and correlated also with infiltration on perirectal tissue (p = 0,05) [9]. In our study found that DIS among well, moderate and poor grade there were significantly different (p = 0,001). The difference between well diff and moderate diff grades showed significant different, but not between moderate diff and poor diff. This can be due to the number of moderate diff samples more than four times amount of the poor diff sample.

In the rectal sample analysis, the results of DIS length in each grading histology (well, moderate and poor) showed no significant difference (p = 0.052). The result of 0.052 can be concluded as just almost significant, which is likely if more samples, it will obtain significant results. This possibility can be reviewed on the correlation test obtained p value = 0.012 indicating that the relation between grading histology with distal intramural spread is positive direction with moderate correlation strength.

Results of data analysis of DIS among stages in colorectal samples showed significant different with p < 0.049. This means that the length of DIS in each stage there was significant different. While result of data analysis of DIS with stadium on rectal samples obtained result no significantly difference, with p = 0,057. The near 0.057 (<0.05) can be summed up as almost significant, which is likely if more samples it will get significant results. This is supported by the results of the correlation test obtained p value < 0.001 indicating that the relationship between DIS and stage is meaningful with the direction of positive correlation and strong correlation strength. With a strong positive correlation it can be interpreted that the longer the DIS will be the higher stage. In the research of Andreola et al. it was found that colorectal cancer patients at Dukes A and Dukes B after microscopically measured had DIS less than 10 mm, whereas in Dukes C stage most of the infiltration had been found in the lymph nodes [9].

Analysis of DIS relationship with CEA in colorectal samples showed a significant correlation (p = 0,024) with moderate positive correlation. While in rectal samples obtained results no significant relationship (p = 0,588). Wood et al. Reported that Grade histology of the tumor was strongly associated with local invasion of tumor cells and lymph node metastases [10]. Theory of matrix metalloproteinase is also called matrixin, reportedly plays a central role in this process. The cancer cell corresponds to its histologic grade produce the MMP-9, MMPs divide and degrade all the structural elements of the extracellular matrix (ECM) so that MMP plays a significant role in both lymphogen and haematogenous metastases. So it affects the levels of CEA in the blood. It is said that high grade tumors are associated with increased penetration of the intestinal wall and increased metastasis in the lymph nodes or distant metastases [14]. It can be assumed that high grade tumors will produce more CEA so that metastatic processes can proceed. This assumption is also supported by data from studies where preoperative serum CEA levels are significantly higher in patients with metastatsis, than those without metastasis [15].

The range distal intramural spread (DIS) length in all colorectal samples was 1,07–11,49 mm. The range of distal resection margin was 15,1–59,44 mm with the result of a distal PA resection free of all tumors. The range of DIS in the rectum was 0,28-10,36 mm. And the range distal resection margin was 16,36-30,0 mm with all distal resection free tumor results.

The limitation of this study is the research sample is still limited to one hospital and the total sample is not large number (26 sample) and The sample collection time is relatively short, which is only 7 months. The advantages of this research are this research is the first in Kariadi General hospital Indonesia and the results of this study can be used as a standard reference for the safety margin of colorectal caesarean resection, especially for Indonesian and Asian people.

## 5 Conclusion

The range of DIS of colorectal samples were between 1.07 and 11.49 mm. This study proved that the longer DIS were occurred when grading histology worsens, tumor stage increases and the higher CEA levels. For the rectal location, the DIS length range were between 0.28 and 10.36 mm. The longer DIS were occurred when grading histology worsens and the tumor stage increases. The DIS length of the rectum was not proven to correlate with elevated CEA levels. Thus, histologic grading, tumor stage and CEA levels can be predictor of distal intramural spread (DIS) colorectal cancer.

The strongest correlation were between DIS and histologic grading. Thus, in mid and lower third of the rectal cancer, the histologic grade examination is strongly recommended. Based on this study, it is recommended that in rectal cancer undergoing sphincter preserving surgery distal resection sould be more than 2 cm from the tumor margin.

## Ethical approval

All procedure for research has been approved by Ethics Commission Faculty of Medicine, Diponegoro University/Dr.Kariadi General Hospital.

## Sources of funding

No funding or sponsorship.

## Author contribution

AJ, IR, and DEL wrote the manuscript and participated in the study design. AJ, IR, DEL, FD and RHA drafted and revised the manuscript. AJ, and IR performed treatment and surgery of colon or rectal adenocarcinoma Patients. AJ, IR, DEL, FD and RHA performed bioinformatics analyses and revised the manuscript. All authors read and approved the final manuscript.

## Trial registry number

1. Name of the registry: http://www.researchregistry.com. Registration Date: February 13, 2021 03:20
2. Unique Identifying number or registration ID: researchregistry6569
3. Hyperlink to your specific registration (must be publicly accessible and will be checked): https://www.researchregistry.com/browse-the-registry#home/registrationdetails/602745772fd2c3001c6c3706/

## Guarantor

Rohadi Muhammad Rosyidi.

## Provenance and peer review

Not commissioned, externally peer-reviewed.

## Consent

This manuscript data from medical record patients diagnosed with colon or rectal adenocarcinoma in the period of September 2017–March 2018 and underwent resection at Dr.Kariadi Hospital.

## Declaration of competing interest

The authors report no conflict of interest.
